# Immunomodulatory Effect of Adipose Stem Cell-Derived Extra-Cellular Vesicles on Cytokine Expression and Regulatory T Cells in Patients with Asthma

**DOI:** 10.3390/ijms251910524

**Published:** 2024-09-30

**Authors:** Jae Hoon Jung, Shin Ae Kang, Ji-Hwan Park, Sung-Dong Kim, Hak Sun Yu, Sue Jean Mun, Kyu-Sup Cho

**Affiliations:** 1 Department of Otorhinolaryngology, Pusan National University School of Medicine, Yangsan 50612, Republic of Korea; narcissism20@naver.com; 2 Department of Environmental Medical Biology, Catholic Kwandong University College of Medicine, Gangneung 25601, Republic of Korea; f2s4u@pusan.ac.kr; 3 Department of Otorhinolaryngology and Biomedical Research Institute, Pusan National University School of Medicine, Pusan National University Hospital, Busan 49241, Republic of Korea; nobleivy@naver.com (J.-H.P.); applekims@hanmail.net (S.-D.K.); 4 Department of Parasitology and Tropical medicine, Pusan National University School of Medicine, Yangsan 50612, Republic of Korea; hsyu@pusan.ac.kr; 5 Department of Otorhinolaryngology and Research Institute for Convergence of Biomedical Science and Technology, Pusan National University Yangsan Hospital, Yangsan 50612, Republic of Korea; baskie23@naver.com

**Keywords:** mesenchymal stem cells, extracellular vesicles, T lymphocytes, regulatory, immunosuppression therapy, asthma

## Abstract

Although mesenchymal stem cell (MSC)-derived extracellular vesicles (EVs) are as effective as MSCs in the suppression of allergic airway inflammation, few studies have evaluated the immunomodulatory capacity of MSC-derived EVs in patients with asthma. Thus, we assessed the effects of adipose stem cell (ASC)-derived EVs on cytokine expression and regulatory T cells (Tregs) in peripheral blood mononuclear cells (PBMCs) of asthmatic patients. PBMCs (1 × 10^6^ cells/mL) were isolated from asthmatic patient and healthy controls and co-cultured with 1 μg/mL of ASC-derived EVs. Th (T helper) 1-, Th2-, and Treg-related cytokine expression, fluorescence-activated cell sorting analysis of CD4^+^CD25^+^FOXP3^+^ T cells, and co-stimulatory molecules were analyzed before and after ASC-derived EV treatment. The expression levels of IL-4 and costimulatory molecules such as CD83 and CD86 were significantly higher in PBMCs of asthmatic patients than in control PBMCs. However, ASC-derived EV treatment significantly decreased the levels of interleukin (IL)-4 and co-stimulatory molecules such as CD83 and CD86 in the phytohemagglutinin (PHA)-stimulated PBMC of asthmatic patients. Furthermore, ASC-derived EVs remarkably increased the transforming growth factor-β (TGF-β) levels and expression of Tregs in the PBMC of asthmatic patients. ASC-derived EVs induce Treg expansion and have immunomodulatory effects by downregulating IL-4 and upregulating TGF-β in PBMCs of asthmatic patients.

## 1. Introduction

Bronchial asthma is a chronic eosinophilic airway inflammation associated with bronchial hyper-responsiveness (AHR), mucus hypersecretion, and airway remodeling [1]. Epigenetic mechanisms such as histone modifications and DNA methylation have been found to play a pivotal role in T cell differentiation and asthma development [2]. Excessive activation of T helper type 2 (Th2) cells by insufficient suppression of regulatory T cells (Tregs) has been thought to play a critical role in the pathogenesis of allergic airway diseases [3,4]. Th2 cell-mediated inflammation is characterized by increased Th2 cytokines (e.g., interleukin (IL)-4, IL-5, and IL-13), high serum immunoglobulin (Ig) E levels, and eosinophilic infiltration in the lung [3,5]. Tregs expressing the transcription factor Foxp3 are known to suppress allergic airway inflammation by secreting anti-inflammatory cytokines such as IL-10, IL-35, and transforming growth factor-β (TGF-β) [6]. Numerous regulatory peptides such as neuropeptides and adipokines play an important role in the pathophysiology of bronchial asthma [7]. Neuropeptide Y and tachykinins exacerbate asthma symptoms, but nociceptin and neurotensin have ameliorating properties [7]. Furthermore, leptin and resistin promote airway inflammation, while adiponectin and ghrelin reduce airway hyperreactivity in obesity-related asthma [7].

Mesenchymal stem cells (MSCs) including those derived from adipose tissue modulate immune responses and inflammation. Several studies have shown that adipose stem cells (ASCs) and other MSCs decreased allergic airway inflammation in bronchial asthma mouse models [8,9,10]. Furthermore, ASC-conditioned media or ASC-derived extracellular vesicles (EVs) were effective as ASCs themselves in improving allergic airway diseases [11,12]. The immunomodulatory effects of ASC-derived EVs in allergic airway inflammation may be mediated by the suppression of Th2 cytokine production and induction of Treg expansion [13,14]. Furthermore, compared with ASC treatment, cell-free therapy mediated by ASC-derived EVs has many advantages including safety, ease of handling or storage, lower possibility of immune rejection, and no risk of vascular occlusion [11,12,15]. Although a recent study showed that ASC-derived EVs improved allergic airway inflammation in a mouse model of asthma, [12] the effects of ASC-derived EVs on Th2-mediated inflammation in patients with asthma remain to be elucidated. 

In this study, we isolated EVs from the conditioned media of murine ASCs and evaluated their immune modulating effect on peripheral blood mononuclear cells (PBMCs) of asthmatic patients compared to healthy control subjects.

## 2. Results 

### 2.1. Characterization of ASC-Derived EVs

**Figure 1:**
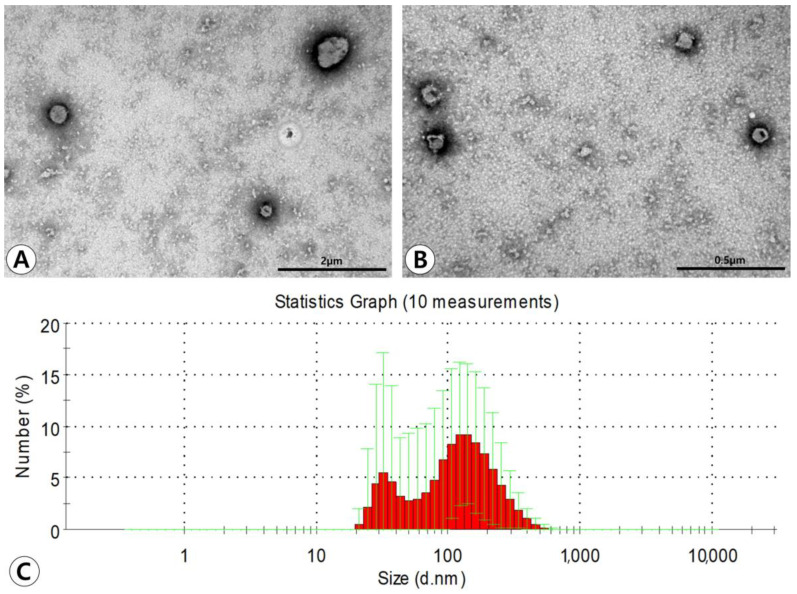
Characterization of adipose stem cell (ASC)-derived extracellular vesicles (EVs). (**A**,**B**) Transmission electron microscopy demonstrated that ASC-derived EVs have lipid bilayers and spherical shape (original magnification ×50,000 and ×80,000, respectively). (**C**) The average size of the EVs measured by dynamic light scattering was 130.3 ± 65.7 nm in diameter. The majority of particles isolated from the ultracentrifugation-concentrated EV pellets were in a size range of approximately 100–200 nm.

Transmission electron microscopy (TEM) demonstrated that ASC-derived EVs had lipid bilayers and were spherical in shape (Figure 1A,B). Western blotting showed that ASC-derived EVs were positive for the CD81 exosome-specific marker and CD40 microvesicle-specific marker. The average size of the EVs measured by using dynamic light scattering (DLS) was 130.3 ± 65.7 nm in diameter. The majority of particles isolated from the ultracentrifugation-concentrated EV pellets were in a size range of approximately 100–200 nm. However, smaller amounts of smaller particles (approximately 40–100 nm) were also observed (Figure 1C). 

### 2.2. Analysis of Cytokine Expression

**Figure 2:**
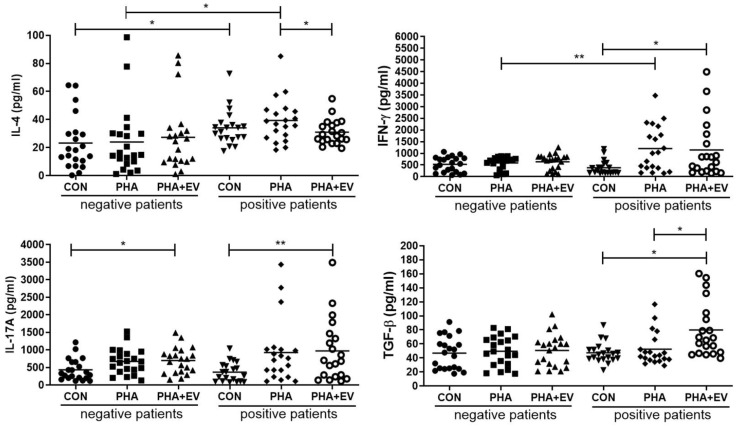
Effects of adipose stem cell (ASC)-derived extracellular vesicles (EVs) on cytokine levels. IL-4 level was significantly higher in the CON and PHA group of asthmatic patients than healthy controls. ASC-derived EVs markedly decreased the levels of IL-4 but increased levels of TGF-β in the PHA group of asthmatic patients. IFN-γ levels were significantly higher in the PHA group of asthmatic patients than healthy controls. ASC-derived EVs significantly elevated the IFN-γ levels in the CON group of asthmatic patients and IL-17A levels in the CON group of healthy controls and asthmatic patients. The horizontal lines represent the means. CON, control; IFN, interferon; IL, interleukin; PHA, phytohemagglutinin; TGF, transforming growth factor. * *p* < 0.05; ** *p* < 0.01.

The expression level of IL-4 was significantly higher in the control (CON) and PHA group of asthmatic patients than in healthy controls (*p* = 0.040 and *p* = 0.022, respectively). However, treatment of ASC-derived EVs markedly decreased the IL-4 level in the PHA-stimulated PBMC of asthmatic patients (*p* = 0.045). The interferon (IFN)-γ level was significantly higher in the PHA group of asthmatic patients than healthy controls (*p* = 0.008). However, ASC-derived EVs remarkably increased the IFN-γ level in the CON group of asthmatic patients (*p* = 0.012). After ASC-derived EV treatment, the IL-17A levels were significantly elevated in the CON group of healthy controls and asthmatic patients (*p* = 0.017 and *p* = 0.006, respectively). Furthermore, ASC-derived EV treatment markedly increased the TGF-β expression in the CON and PHA group of asthmatic patients (*p* = 0.001 and *p* = 0.011, respectively) (Figure 2).

### 2.3. Expression of CD4^+^CD25^+^FOXP3^+^ T Cells

**Figure 3:**
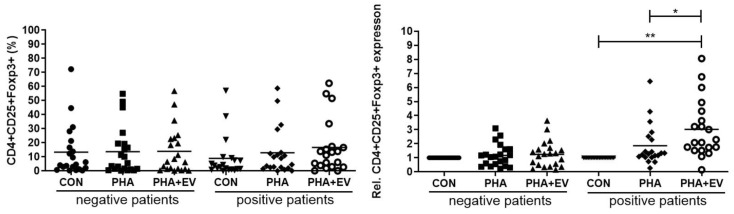
Effects of adipose stem cell (ASC)-derived extracellular vesicles (EVs) on CD4^+^CD25^+^FOXP3^+^ T cells. Although there were no significant differences in the percentage of CD4^+^CD25^+^FOXP3^+^ T cells among the groups, ASC-derived EVs significantly increased the relative expression of CD4^+^CD25^+^FOXP3^+^ T cells in the CON and PHA group of asthmatic patients. The horizontal lines represent the means. CON, control; PHA, phytohemagglutinin, Rel, relative. * *p* < 0.05; ** *p* < 0.001.

In the healthy controls, the populations of CD4^+^CD25^+^FOXP3^+^ T cells were not significantly different after PHA stimulation or ASC-derived EV treatment. Although there were no significant differences in the percentage of CD4^+^CD25^+^FOXP3^+^ T cells among the groups, ASC-derived EV treatment significantly increased the relative expression of CD4^+^CD25^+^FOXP3^+^ T cells in the CON and PHA group of asthmatic patients (*p* < 0.001 and *p* = 0.046, respectively) (Figure 3).

### 2.4. Analysis of Costimulatory Molecules on Monocytes

**Figure 4:**
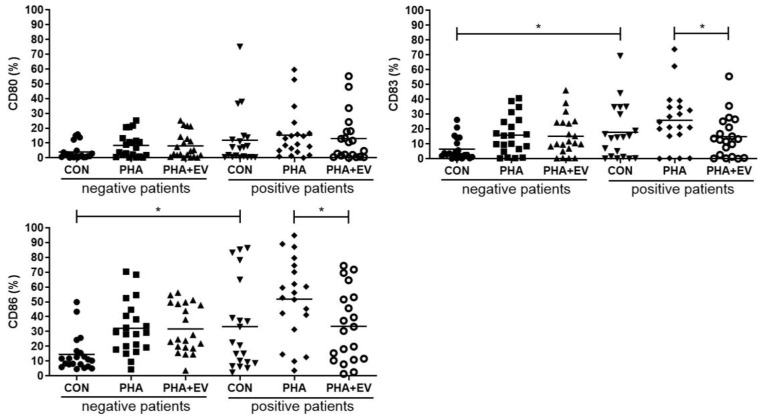
Effects of adipose stem cell (ASC)-derived extracellular vesicles (EVs) on CD80, CD83, and CD86. The percentages of CD83 and CD86 were significantly higher in the CON group of asthmatic patients than healthy controls. ASC-derived EVs significantly decreased the percentage of CD83, and CD86 in the PHA-stimulated PBMC of asthmatic patients. The horizontal lines represent the means. CON, control; PHA, phytohemagglutinin. * *p* < 0.05.

The percentage of CD83 and CD86 were significantly higher in the CON group of asthmatic patients than in healthy controls (*p* = 0.013 and *p* = 0.012, respectively). The expression of CD80, CD83, CD86 was significantly increased after PHA stimulation. However, ASC-derived EV treatment significantly decreased the percentage of CD83 and CD86 in the PHA-stimulated PBMC of asthmatic patients (*p* = 0.048 and *p* = 0.029, respectively) (Figure 4).

## 3. Discussion

MSC-derived EVs have been reported as promising candidates to treat allergic airway diseases because they can modulate immune function [16,17]. Several previous studies have shown that MSCs themselves or MSC-derived EVs decrease airway eosinophilic infiltration, allergic-specific Th2 cytokines, IgE production, and AHR in an ovalbumin-induced asthmatic mouse model [8,9,11,12]. Furthermore, ASC-derived EVs ameliorated Th2-mediated inflammation through the upregulation of IL-10 and TGF-β and the downregulation of eotaxin and IL-25 in mouse lung epithelial cells, accompanied by M2 macrophage polarization and dendritic cell activation [13]. MSC-derived EVs may have immunomodulatory effects by delivering microRNAs (miRNAs) and increasing pulmonary genes such as paraoxonase-1 and secretoglobin family 1C member 1, which lead to the expansion of Tregs [14,18,19]. However, there are no studies that evaluate the specific roles of MSC-derived EVs in patients with asthma. To our knowledge, this is the first study to investigate the immunomodulatory effects of MSC-derived EVs on cytokine expression, Tregs differentiation, and costimulatory molecules in PBMCs of asthmatic patients.

EVs are spherical bi-layered proteolipids secreted by almost cell types such as hematopoietic cells, endothelial cells, cancer cells, and MSCs under physiological or pathological conditions [20,21]. MSC-derived EVs can exert their effects by delivering their contents, which could be proteins, mRNAs, or miRNAs, to recipient cells [22]. They can be classified into three subgroups according to their origin, biogenesis, shape, composition, and diameter: exosomes (30–100 nm), microvesicles (100–1000 nm), apoptotic bodies (1000–5000 nm) [23]. Previous studies have shown that mouse MSCs share the same morphological and functional characteristics as human MSCs [24,25]. Furthermore, human MSC-derived EV procurement has numerous potential downsides including pain, donor site morbidity, and ethical problems about human experiments. Therefore, we used mouse ASC-derived EVs because of their abundance, easy harvesting, and avoidance of ethical concerns. The ASC-derived EVs used in the present study were characterized by heterogeneously spherical bodies using TEM, with diameters ranging from approximately 30 nm to 300 nm, consistent with a mix of exosomes and microvesicles. Furthermore, western blotting analysis demonstrated high levels of the CD81 exosome-enriched marker and CD40 microvesicle-enriched marker.

In this study, the expression levels of IL-4 and costimulatory molecules such as CD83 and CD86 were significantly higher in PBMCs of asthmatic patients than in control PBMCs. IL-4 is a central Th2 cytokine in the development of allergic inflammation with distinct roles in the induction of the ε isotype switch, secretion of IgE by B lymphocytes, and eosinophil trafficking [26]. The costimulatory molecules, CD80, CD83, and CD86, are type I integral membrane glycoproteins that are expressed on the surface of activated T lymphocytes, B lymphocytes, and antigen-presenting cells (APCs) [27]. Among costimulatory molecules, CD83 has the function of promoting the expression of other activation markers such as CD86 and the major histocompatibility complex (MHC) class II [28]. As Th1 and Th2 cell responses are mutually antagonistic in the development of asthma, selective suppression of Th2 cytokines may be crucial for protection against allergic airway inflammation [3]. The present study demonstrated that ASC-derived EVs significantly alleviated Th2 response in asthmatic patients. Treatment of ASC-derived EVs significantly lowered the levels of IL-4, CD83, and CD86 in the PHA-stimulated PBMC of asthmatic patients. However, there was no significant difference in the level of IFN-γ or IL-17A before and after ASC-derived EV treatment. These data are consistent with previous studies that ASCs or ASC-derived EVs attenuate allergic airway inflammation in a mouse model of asthma [8,9,10,11,12,13,14,15].

PBMCs include CD3^+^ T cells, B cells, natural killer cells, monocytes, and dendritic cells. The CD3^+^ T cells are composed of CD4^+^ and CD8^+^ T cells. After activation with CD3/CD28 T cell activator, the CD4^+^ T cells may develop into diverse effector cell subsets, including Th1, Th2, Th17, Th9, Th22, and Tregs [29]. Tregs, characterized by CD4, CD25, and transcription factor Foxp3, are a unique T cell population with strong immune suppressive properties [30]. The suppressive function of induced Tregs is mediated by the production of anti-inflammatory cytokines such as IL-10 and TGF-β [31]. The immunomodulatory effects of ASCs themselves or ASC-derived EVs in allergic airway inflammation could be mediated by the upregulation of Tregs and the increased expression of IL-10 and TGF-β [8,12,14]. Consistently, our study showed that ASC-derived EVs significantly increased the TGF-β levels and expression of Tregs in the PBMC of asthmatic patients. Our findings, and those from other reports, provide evidence that Treg expansion plays a critical role in the immunomodulatory properties of ASC-derived EVs. Although the immunomodulatory mechanism of ASC-derived EVs in PBMCs of asthmatic patients remains unclear, the EV-mediated miRNA transfer including miR-1470, miR-146a-5p, miR-126-3p, and miR-301a-3p may be responsible for downregulating IL-4, upregulating TGF-β, and inducing Treg expansion [14,32]. Furthermore, the plasma EV miR-17-92 and miR-106a-363 clusters were linked to inflammatory and metabolic mechanisms of obesity-associated low type-2 asthma [33].

Our study has some limitations. T lymphocyte numbers and cytokine expression isolated from PBMCs of asthmatic patients did not proliferate without PHA. Therefore, there was no significant difference before and after ASC-derived EV treatment in the absence of PHA. Because in vitro studies do not always reflect the in vivo situation, the immunosuppression capacity of ASC-derived EVs in asthmatic patients should be evaluated in the future. There may be some differences in EV transmitters among healthy, asthmatic, and obese humans. Further study should compare the plausible role of ASC-derived EVs according to the human subjects. Furthermore, molecular and genetic research may also be required to elucidate the immunomodulatory mechanism of ASC-derived EVs in asthmatic patients.

## 4. Materials and Methods

### 4.1. Subjects 

A total of 21 asthmatic and 21 healthy control adults aged between 20 and 69 years were enrolled in this study. Patients diagnosed with asthma were aged 24 to 69 years, with a mean age of 51.6 years (12 males, 9 females). Healthy subjects were aged 20 to 67 years, with a mean age of 66.4 years and 50.1 years (14 males, 7 females). Bronchial asthma was confirmed by a respiratory physician based on medical history and supported by evidence of variable airflow obstruction or airway hyperresponsiveness (AHR) using a methacholine challenge test. Healthy subjects were asymptomatic and had no known respiratory illness, with normal spirometry results. The characteristics of asthmatic patients and healthy controls are summarized in Table 1. Patients with a known immunodeficiency, chronic renal failure, bleeding disorders, anticoagulant therapy, or who had received a systemic corticosteroid during the previous one month were excluded. This study protocol was approved by the Institutional Review Board of Pusan National University Yangsan Hospital (04-2109-003) and performed in accordance with relevant guidelines and regulations.

### 4.2. Isolation and Culture of ASCs

Among MSC, ASCs were used because of their abundance, relatively easy harvesting, and high proliferation potential. Adipose tissue was obtained from the abdominal fat of C57BL/6 mice according to previous reports [8,18]. To isolate homogenous ASCs, adipose tissue was digested with 0.075% collagenase type I (Sigma-Aldrich, St. Louis, MO, USA) at 37 °C for 30 min after washing extensively with phosphate-buffered saline (PBS). After neutralization of enzyme activity with α-modified Eagle’s medium (α-MEM) containing 10% fetal bovine serum (FBS), the sample was centrifuged at 1200× *g* for 10 min to obtain a pellet. The pellet was filtered through a 100 μm nylon mesh to remove cellular debris and incubated overnight at 37 °C in 5% carbon dioxide (CO_2_) in control medium containing α-MEM, 10% FBS, 100 unit/mL penicillin, and 100 μg/mL streptomycin. After incubation, residual non-adherent cells were removed by washing extensively with PBS. One week later, when the monolayer of adherent cells had reached confluence, cells were trypsinized, resuspended in α-MEM containing 10% FBS, and subcultured at the concentration of 2000 cells/cm^3^. Flow cytometric analysis was performed to characterize the phenotype of the ASCs. Third or fourth passages of ASCs were used for the experiments. 

### 4.3. EV Extraction and Characterization

ASCs were cultured at 37 °C with 5% CO_2_ in α-MEM containing 10% FBS until 1 × 10^6^ cells/cm^2^ were obtained. After centrifugation at 12,000× *g* for 30 min, the culture supernatant of ASCs was collected and freeze-dried. The unnecessary excessive salts in the ASC-conditioned media were removed by HiTrap^®^ Desalting Columns (Cytiva, Uppsala, Sweden). EVs were isolated from ASC-conditioned media as previously described [12,13,18]. The supernatant was filtered through a 0.45 μm vacuum filter. The filtrate was concentrated using a QuixStand (GE Healthcare, Little Chalfont, UK) and then filtered through a 0.22 μm bottle top filter (Sigma-Aldrich, St. Louis, MO, USA). The filtrates were pelleted by ultracentrifugation in a 45 Ti rotor (Beckman Coulter, Fullerton, CA, USA) at 100,000× *g* for 2 h at 4 °C. The final pellets were resuspended in PBS and stored at −80 °C. We placed the EVs in PBS on 300-mesh copper grids and stained them with 2% uranyl acetate. Images were obtained using a JEM-1011 electron microscope (JEOL, Tokyo, Japan) operated at an acceleration voltage of 100 kV. EV markers including CD81 and CD40 were analyzed by western blotting with primary antibodies, anti-CD81 (1:1000, Abcam, Cambridge, MA, USA), and anti-CD40 (1:1000, Abcam, Cambridge, MA, USA) as previously described [13]. To analyze CD81 and CD 40 expression, 30 μg of EV was separated by Mini-PROTEAN TGX Gels (Bio-Rad, Hercules, CA, USA) electrophoresis at 100 V for 90 min. The separated proteins were transferred onto polyvinylidene difluoride membranes (Amersham Biosciences, Amersham, UK), and the membranes were blocked overnight with 5% skim milk in Tris-buffered saline containing 0.1% Triton X-100 (Sigma-Aldrich, St. Louis, MO, USA). The membranes were washed five times and incubated with anti-CD81 (rabbit monoclonal Ab, # D502Q) (Cell Signaling Technology, Danvers, MA, USA) and anti-CD40 (rabbit polyclonal Ab, ADI-CSS-180) (Enzo Life Sciences, Farmingdale, NY, USA), diluted at 1:1000 in the blocking buffer, overnight at 4 °C. Detection was performed with horseradish peroxidase-conjugated secondary anti-rabbit antibodies used at a 1:1000 dilution for 1 h at room temperature. The blot for horseradish peroxidase was developed using the ECL substrate (Amersham Biosciences, Amersham, UK). The diameters of EVs were measured using a Zetasizer Nano ZS (Malvern Instruments Ltd., Worcestershire, UK) equipped with a 633 nm laser line at a scattered intensity of 10 × 30 s. 

### 4.4. Isolation of PBMCs and Co-Culture with ASC-Derived EVs 

Peripheral blood samples were collected from asthmatic patients and healthy controls. Blood samples were diluted 1:1 with sterile PBS containing 2% FBS, then transferred over Ficoll-paque solution PLUS (GE Healthcare, Uppsala, Sweden) in a SepMate 50 mL tube for density gradient separation. After centrifugation at 1200× *g* for 10 min at room temperature, the top layer containing the enriched mononuclear cells (MNCs) was transferred into a new tube. The enriched MNCs were washed twice with PBS containing 2% FBS and counted. PBMCs (1 × 10^6^ cells/mL) were cultured in RPMI/5% human AB serum with or without (control) phytohemagglutinin (PHA) at a final concentration of 15 μg/mL by stimulating with human CD3/CD28 T cell activator (STEMCELL Technologies, Vancouver, BC, Canada) in 24 well plates [34]. PBMCs from asthmatic patients and healthy controls were cultured for 72 h with or without 1 μg/mL ASC-derived EVs. After 3 days, culture supernatants were collected for cytokine measurement, and cells were harvested for flow cytometric analysis [34,35].

### 4.5. Detection of Cytokine Levels 

After 3 days of ASC-derived EV treatment, IL-4, IL-17A, IFN-γ, and TGF-β levels in the culture supernatant were determined by sandwich enzyme-linked immunosorbent assay (ELISA) (eBiosciences, San Diego, CA, USA) in accordance with the manufacturer’s recommendations. Microtiter plates (Costar, Cambridge, MA, USA) were coated with the capture antibody at 4 °C overnight. After blocking with 2% bovine serum albumin in PBS for 1 h, undiluted supernatant was added and incubated for 2 h at room temperature. 

Subsequently, the cytokine levels were detected with a biotin-conjugated detecting antibody and developed with horseradish peroxidase-conjugated streptavidin and tetramethylbenzidine as a substrate (Endogen, Rockford, IL, USA). The absorbance of the final reactant was measured at 450 nm using an enzyme-linked immunosorbent assay plate reader (Molecular Devices, Sunnyvale, CA, USA).

### 4.6. Phenotypic Analysis of CD4^+^CD25^+^FOXP3^+^ Treg Cells

PBMCs were washed with PBS and incubated at room temperature for 15 min with Fixable Live/Dead Blue Viable Dye (Thermo Fisher Scientific, Loughborough, UK). After incubation, the cells were washed in PBS buffer containing 0.5% bovine serum albumin (BSA) (Sigma-Aldrich, St. Louis, MO, USA) and 2 mM ethylenediamine tetraacetic acid (EDTA) (Sigma-Aldrich, St. Louis, MO, USA). The cells were washed with FACS Buffer and the cell pellet was labelled with a human FOXP3 kit (eBioscience, San Diego, CA, USA) according to the manufacturer’s instructions. Briefly, cells were incubated with anti-CD4-FITC (eBioscience, San Diego, CA, USA) and anti-CD25-APC (eBioscience, San Diego, CA, USA) antibodies for 30 min at room temperature in the dark. After incubation, cells were washed and incubated with Foxp3 Fixation/Permeabilization working solution at 4 °C for 30 min in the dark, and then permeabilized cells were incubated with anti-FOXP3 PE antibody(eBioscience, San Diego, CA, USA) for 40 min at room temperature by avoiding cells from light. After the washing step, supernatant was discarded, and cell lysate was analyzed using a FACSCalibur flow cytometer. Furthermore, the relative expression level of the CD4^+^CD25^+^FOXP3^+^ T cell was based on the expression ratio of the PHA stimulation group or the EV treatment group versus the control group.

### 4.7. Analysis of Costimulatory Molecules on Monocytes

After, PBMCs were left untreated (as a control) or were stimulated with 15 μg/mL of PHA or 1 μg/mL of ASC-derived EVs for 72 h. PBMCs were stained with anti-CD14 APC (eBioscience, San Diego, CA, USA) to distinguish populations of monocytes. Additionally, cells were stained with anti-CD80 FITC (eBioscience, San Diego, CA, USA), anti-CD83 PerCP-eFlour710 (eBioscience, San Diego, CA, USA), and anti-CD 86 PE (eBioscience, San Diego, CA, USA) and analyzed by flow cytometry.

### 4.8. Statistical Analysis 

All experiments were performed three times and then similar results were produced each time. Data were expressed as mean ± standard deviations. The Student’s t test was used to determine group differences, and all statistical analyses were conducted using the SPSS for Windows software package (version 28.0; SPSS Inc., Chicago, IL, USA). Graphs were generated using GraphPad Prism software (version 10.2.3; GraphPad Software, Inc., SanDiego, CA, USA). A value of *p* < 0.05 was considered to indicate statistical significance.

## 5. Conclusions

ASC-derived EVs promote Treg expansion and have immunomodulatory effects by upregulation of TGF-β and downregulation of IL-4 in PBMCs of asthmatic patients. Therefore, ASC-derived EVs may be useful as a new promising therapeutic candidate for the treatment of asthma. 

## Figures and Tables

**Table 1:** Characteristics of asthmatic patients and healthy controls.

Characteristics	Asthmatic Patients	Healthy Controls
Age, years	51.6 ± 13.6	66.4 ± 12.3
Male, n (%)	12 (57.1)	14 (66.7)
BMI, kg/m^2^	23.3 ± 2.9	23.7 ± 5.6
Serum IgE, IU/mL	852.3 ± 734.1	89.2 ± 23.5
Eosinophil, %	6.4 ± 4.6	3.1 ± 3.0
Allergic skin test (+), n (%)	14 (66.7)	7 (33.3)
FEV1, % predicted	82.4 ± 24.1	104.1 ± 4.5
FEV1/FVC, %	68.0 ± 15.2	79.8 ± 2.2
FeNO, ppb	77.0 ± 54.9	24.1 ± 7.8

Data are expressed as means ± standard deviation except male, allergic skin test (number (percentage)). BMI, body mass index; Ig, immunoglobulin; FEV1, forced expiratory volume in one second; FVC, forced vital capacity; FeNO, fractional exhaled nitric oxide.

## Data Availability

Data is contained within the article.
